# The Buffalo Pathological Society

**Published:** 1889-12

**Authors:** 


### THE BUFFALO PATHOLOGICAL SOCIETY.

REGULAR MEETING, NOVEMBER 15, 1889.

The President, Dr. ROSWELL PARK, in the chair.

Dr. IRVING M. SNOW brought an old lady suffering from the rare condition of word blindness.

Dr. PUTNAM and Dr. KRAUSS made several tests at Dr. Snow's request, demonstrating the conditions mentioned in the report.

Dr. WM. C. KRAUSS then presented the following case:

Male, aged 34 years; painter by occupation. No history of syphilis, alcoholism, or neuropathic taint.

August 23, 1889, while at work on a scaffolding sixteen feet high, was suddenly precipitated to the ground, and received a contusion over the left temporal fossa. Was awakened the following morning by a hissing sound in his head. An examination showed it to be an aneurism of the left internal maxillary artery, based on the following symptoms: Pulsation, slight swelling and redness over the left temporal fossa; the presence of a bruit, regular in rhythm and intensity, synchronous with the apex beat, which disappears on pressure over this region. Cessation of the bruit by pressure on the left external carotid artery, at the inferior posterior angle of the inferior maxillary bone; no modification by pressing on the temporal artery. The treatment thus far has been ergot, iodide of potash, light cathartics, and abstention from excesses. For radical treatment, it is proposed to ligate the external carotid artery at the inferior posterior angle of the jaw.

After the reading of the minutes, the paper ot the evening was presented:

### ANNUAL ADDRESS OF THE PRESIDENT.

BY ROSWELL PARK, M. D.

It has been my deliberate attempt for this evening to establish a precedent which, I trust, you will insist that my successors in office shall follow: that is, to make an informal address to you upon some subject in pathology, or the cognate branches, at the conclusion of this term of service. I do this with a conviction that it is to the best interests of our Society. Yet, before I begin with the topic to which I solicit your attention this evening, permit me a few rambling sentences. I desire heartily to congratulate the members of this Society upon the work accomplished during the first year of its existence.

It has been always of a dignified and elevated character, and would have been creditable to any society in this country. I have heard papers of vastly lower standard than those presented to you in organizations whose names are everywhere known, and whose publications are widely read. You have certainly done the best work in this part of the State, since we began working together, and I am intensely in earnest when expressing a hope that your membership may steadily increase, and that your interest in its objects and its meetings may never flag.

I should, moreover, be unjust to myself and ungrateful to you, did I not acknowledge the great compliment you did me when you made me your first presiding officer, and confess that it has been with a peculiar pride that I have endeavored to act in that capacity. I trust that you may have many more energetic, even enthusiastic, presidents, but I am sure you will never have a more devoted one.

It has been somewhat difficult for me, as a specialist, to find a topic which we all possess in common, as it were, and which might appeal to each of you with something like equal interest. On reflection, it occurred to me that we all, alike, no matter what our specialty or our practice, have to contend in one way or another with that arch-enemy *pus*.

Pus, like our next door neighbor, is seen so often that we pay too little attention to it, yet we cannot overestimate the importance of a better acquaintance with it than we enjoy to-day. This, then, is my theme to-night, for which I crave, if need be, your indulgence.

What is pus? A few years ago this question was comparatively easily answered. That it is now a query to which it is extremely difficult to give an explicit answer, is simply an evidence of progress in the study of pathology. A former and revered teacher used to express it tersely that "pus is dead or dying blastema." Even the term blastema is now almost obsolete. According to Robin, *blastema* means "the substance resulting from the elaboration of nutritive material furnished to the anatomical elements by the blood." Foster's dictionary gives as other definitions: "Undifferentiated embryonic tissue; the material out of which a part is to be formed; and, "a free or parenchymatous plastic exudate." These definitions are sufficiently succinct to indicate that dead or dying blastema must be good and valuable material going or gone to the bad. In a rough and off-hand way, therefore, this conception of the term *pus* may be considered sufficient as a working basis for a further study of the substance itself.

But, as generally used by the clinician, the term is applied alike to the contents of acute or cold abscesses, which have never known exposure to the air, to the discharge from mucous, as well as granulating surfaces, and to the fluid or semi-solid results of degeneration of various tissues. Are these various substances identical, and do they deserve the same name? This is a vexed subject in the domain of surgical pathology, to a discussion of which this paper is in the main devoted.

Many and many a time I have seen my operation wounds heal by primary union, under an aseptic dressing. Of them I could say, as we usually do under such circumstances, they healed without suppuration. And yet, if the drainage-tube—supposing one to have been used—had been left *in situ* a few hours too long, there would be found about its opening, or in its lumen, a drop, perhaps a few drops, of creamy, semi-solid material, which we would ordinarily call pus. Is this material identical with the pus from an acute abscess? To this inquiry I have devoted no small time and study, both at the desk and in the laboratory, and such conclusions as I have reached shall appear further on.

There is a popular expression: "There are dogs and dogs." Can we not also say, "There is pus and pus?"

This subject can only be approached by a careful study of the gross and minute appearances of pus, and the circumstances under which it is produced. A study by which these questions may be answered is inseparable from a study of inflammatory phenomena, with which I must then, for a little while, detain you.

Virchow has made this distinction between physiological and pathological irritation (*Reiz*), that in the former case the function of the cell, or the collection of cells (the organ), is simply increased; in the latter it is disturbed. The entire process by which an alteration or disturbance of nutrition is thus brought about by irritation, he considered to be a *progressive process*, but not necessarily an inflammation. It might result in hyperplasia (numerical cell-increase), inflammation, or tumor formation. He classified irritations as mechanical, chemical and physical, *i. e.*, thermic and electrical. Only such irritations as lead to inflammation interest us here, and, as we shall see, in considering pus formation we shall have to practically limit ourselves to a consideration of microörganisms as the sole causes of such irritation.

At that time (1870) Virchow distinguished *four* degrees of the inflammatory process:

1. A form distinguished—aside from changes in the cells themselves—by watery, serous, albuminous, or mucinous exudate.
2. A form in which the exudate is fibrinous (croupous).
3. A form in which pus is produced.
4. A form characterized by hemorrhagic exudate.

He considered these as progressive stages of one and the same kind of irritation, belonging to either of the three classes before named.

Applied to the study of the repair of wounds, this doctrine taught that mechanical irritation (which caused them) alone was sufficient to explain the formation of pus, that it was unnecessary to seek further for its cause, and that tumefaction of the wound edges bears the same relation to failure to secure primary union that suppuration does to healing by granulation. This opinion seemed the more plausible, since the wounds whose borders presented least tumefaction, were those which healed most kindly *per primam.*

Attack upon this doctrine was speedy and determined. Cohnheim had published in 1867 his studies of the diapedesis of the leucocytes, and the importance of this publication, as well as its accuracy, were almost universally recognized; while the leading part heretofore played by the connective tissue corpuscle, according to Virchow's views, had now, at least, to be shared by the wandering leucocyte. This opinion has been since strengthened, to the point of conviction, by the labors of Cohnheim, Ziegler, and their scholars, so that now it is possible to find an explanation for such neoplasms as belong to the category of inflammatory, regenerative, hyperplastic, or callus, in the known properties of the leucocyte.

I say it is now *possible,* even *probable,* but hold that even yet we are not in position to go to extremes. Cohnheim's enthusiastic followers claim that Virchow has considered innumerable cells to be descendants of connective tissue corpuscles, which are, in fact, escaped leucocytes. Even granting this, there has been no sufficient evidence yet adduced to show that the connective tissue cells are necessarily or absolutely passive, and take no part in cell proliferation. Consequently, it seems as if, in this controversy, the middle ground is certainly the safer.

But the attacks upon Virchow's dicta were made not alone by the histologists, but by those who, like Klebs, contested them upon etiological ground. By 1872, in Germany, the Listerian system had been pretty well adopted, and there no longer remained a doubt but that wound suppuration was caused by contamination of instruments, fingers, dressings, etc., with bacteria. Other infectious inflammations, *e. g.,* endocarditis and erysipelas, were correctly ascribed to microbes, and, in 1878, appeared Koch's masterly work on Wound Infection. Now, the importance of mechanical, chemical, and physical irritations, as agents producing suppuration, was lost in the overwhelming magnitude of the freshly studied "specific reaction (suppuration) due to a specific virus." To be sure, Virchow retorted, in 1880, that we did not know the exact nature of this specific reaction, and that it must be either chemical or mechanical, which is undeniable; yet it is equally undeniable that bacteria did not figure as irritants, when he so fully discussed the causes and consequences of inflammation, and that he remains to-day rather a sceptic as to some of the new teachings in this respect.

The introduction of the antiseptic method has effected both a revolution and a revelation. It was till lately held that the bacteria of putrefaction were also at the same time the pyogenic. In 1881 Virchow and his scholars claimed that suppuration was not invariably produced by microörganisms, and by them alone, but that when it displayed a milder form, less progressive, it was brought about by purely mechanical causes,—fractures, wounds, etc. But the researches of Ogston, Rosenbach, Passet, and numerous other close and diligent observers, clearly demonstrated that suppuration has but one cause, that it is of parasitic origin, and that the pyogenic bacteria are not to be confounded with the saprogenic or putrefactive.

Studies directed especially to the elucidation of these hotly-disputed questions resulted in unexpected advance. Strassburger, Flemming, and others found that the nucleation which precedes cell proliferation afforded an interesting subject by itself, and karyokinesis is now a well-recognized link in the chain of cell-progression. Not alone in the leucocytes is the karyokinetic process known; it has been studied in the connective tissue cells by Scheltema, Grawitz, and Ribbert. This fact lends additional argument in favor of a position midway between the extremes of Virchow and Cohnheim. Whether the inflammatory irritant acts primarily upon the connective tissue elements, the capillary vessels, the muscular and the fatty tissues, whereby active hyperemia and diapedesis of leucocytes are excited, or whether the reverse is true, will depend upon whether one sides with Virchow in the former case, or with Cohnheim and Weigert in the latter.

According to the views of the humoral pathologists, of whom Rokitansky was the father, pus corpuscles, which were seen in the exudate known to have left the vessels, were supposed to have originated from it, hence the definition of their day—pus is dead blood. The ultimate cause of inflammation and suppuration were sought in the chemical condition of the blood; and the dyscrasiæ, or varieties of badness of the blood, were hence considered the causes of these phenomena. Although the old humoral pathology is now abandoned, it will be seen that it, nevertheless, took cognizance of certain truths, since such dyscrasiæ as diabetes, syphilis, and gout are well known to be predisposing causes of inflammation, though not, *per se*, of suppuration.

It was Virchow who decently interred this humoral doctrine, by showing that the formation of cells out of such exudates alone was impossible. By establishing the dictum *ominis cellula e cellulo,* he founded the new cellular pathology, which was to medical science what Keppler's laws were to astronomy. Proliferation of cells now accounts for all tissue changes, either constructive or degenerative, though, by itself, it fails to supply all the knowledge of causes for which we earnestly yearn.

The misinterpretation of certain cellular phenomena by the cellular pathologists has been, in great measure, atoned for by the discoveries of Cohnheim and his pupils, who repeated, in every possible way, the observations first made in 1848 by Waller and Wallace, and who not only established the fact of the diapedesis of the leucocytes, but showed the vast importance of this process in explaining inflammatory action. If they, in their enthusiasm, claimed for their observations a solution of the whole question, they simply showed themselves human, and so liable to err.

While we are not, even to-day, in position to do more than calmly survey the fields where the pathologists of the recent past have excitedly contended for the accuracy of their own notions, yet we must admit that somewhat of truth was contained in the humoral doctrine, and that Virchow and Cohnheim are both right and both wrong; wrong, however, only in each trying to explain everything upon his own discoveries, and in refusing to see as much of truth in the teachings of his opponent as in his own.

After it was definitely understood that all surgical suppurations were of a parasitic origin, an effort was made to establish for the bacteria which caused them a property *sui generis,* as if they were neither chemical nor mechanical irritants, but possessed some hitherto unknown power. Such a theory prompted the investigations at once set on foot, during 1885–'6, and conducted with most painstaking diligence by Hüter, Rosenbach, Orthmann, Lutton, and numerous others, to be referred to again, by which it was demonstrated that pure or sterile chemicals alone could never produce suppuration. Scheurten, Klemperer, Strauss, and others, have repeated these demonstrations, and have made conviction certain, that *without bacteria or their products, suppuration never occurs.* Some difficulty and confusion have arisen from the fact that it was found, in prosecuting these studies, that certain bacteria were pyogenic in the tissues of one animal, and not in those of another. Thus, Grawitz and Dieckerhoff described a bacillus which thus varied in its effects according to the animal used. In 1887, Grawitz and de Bary showed that very weak dilutions of pure cultures of the pyogenic bacteria (1 to 100, etc.,) were resorbed without provoking suppuration. The daily use of ordinary solutions for hypodermic use is simply a homely illustration of this fact. They further showed that such active fluids as turpentine and strong nitrate of silver solution, which are of themselves actively parasiticide, when used upon certain animals in certain amounts, produced a fluid resembling pus. This fluid, however, contained no bacteria, lacked all the septic or infectious properties of true pus, and was produced under conditions such as never obtain, save in the laboratory of the experimenter, and at his pleasure only, at the expense of extreme precaution.

This is an appropriate place at which to stop, *en passant*, and ask whether it is fair to call such a fluid pus? Its like is not met with clinically, and the pus which we daily meet with, and which causes us so much trouble, is the pus which we particularly study, and which is particularly deserving of the name.

Moreover, Grawitz and Scheurlen have both shown that, after the injection of Brieger's *cadaverin*, the same puruloid fluid is, or may be produced, lacking again all the specific properties of pus. It is, furthermore, quite probable that other ptomaines beside cadaverin, all of which are of bacterial origin, may be found to have a similar effect; though several, at least, have failed so far to evince it. Let it be well emphasized just here, however, that even these few substances which thus have been shown capable of producing this puruloid material, do so only under most favorable conditions of time, duration, quantity, and species of animal used for experiment. Weak ammoniacal and cadaverin injections are resorbed; those of greater strength are followed by watery or albuminous infiltrations, or, sometimes, by exquisite fibrinous exudates; used still stronger, they cause hemorrhage and this pseudo-suppuration; and, finally, when used in full strength, necrosis and gangrene are the consequences. It seems to me, upon both theoretical and experimental grounds, that this puruloid fluid, to which I have above alluded, may be properly considered the product of the death of the cells, resulting from the inflammation set up as the result of the injection of the irritant, *and that it is entitled to be considered pus only in the sense that it is dead blastema*, whereas we all know that the pus with which surgeons meet and contend is something more than dead or even dying blastema; that it contains, at least when active and septic, or infectious, living and lively organisms, whose activity and properties are most pernicious. Here is beautifully demonstrated the accuracy of one of Virchow's observations, which were, in the main, brilliant and comprehensive, that tissue reactions or changes are not characterized by wide distinctions; that pus-production is not to be considered, by itself, as a distinct process, but only as a stage in the various possible inflammatory changes in connective tissue. Adopting this view, we see that the differences between the formation of this puruloid fluid and of pus, consist, in a pathological sense, in the penetration into the tissues of destructive germs, and, in a clinical sense, in the overwhelming pathogenic importance which the tissues and the purulent material now acquire by virtue of *their* presence and poisonous capabilities.

In clinical evidence of this feature, let me adduce the difference between an acute and a cold abscess. In the former, the bacteria are still alive and actively producing poisonous material, in proof of which we have fever, sepsis, local destruction, even death. In the latter case, nature has thrown up a sanitary cordon around the infected area in the shape of a thick investing membrane, the so-called but misnamed *pyogenic* membrane; inside of which the pyogenic bacteria have finally perished from starvation. These cold abscesses persist for months, even years, and may slowly disappear by well-known changes, while the patient presents few, perhaps no signs of fever, sepsis, nor any trouble. In other words, so long as bacteria can live and migrate, the fluid in which they disport themselves is pus, true pus; the fluid of an old, cold abscess is, according to this view, no longer pus. It was pus once; it is now *puruloid* in a second sense.

I have tried often to make cultures of pyogenic bacteria from this material, and failed, for reasons just stated; so have many other observers failed, and our position in this matter is indisputably the correct one.

The conspicuous difference between the teaching of 1871 and that of to-day obtains in this, that the degree of inflammatory disturbance necessary for the production of pus is *not* produced by mechanical nor thermal lesions alone, nor by even chemical irritants, except under most peculiar conditions. All suppurations met with in practice are due to bacterial agency, but mainly when, through this agency, nourished within the tissues or planted upon absorbent wound surfaces, they propagate themselves and give forth their peculiar chemical products, *i. e.*, ptomaines. Still, even then, without some predisposing lesion or condition in animals and men, in tissues capable of resorption, the commonly known pyogenic cocci are innocuous.

To this fortunate fact it is due that not every wound suppurates which is not immediately provided with an antiseptic dressing.

While there is, virtually, no pus without bacteria, the reverse is not necessarily true; for we may have even pyogenic cocci present in relatively very small numbers without formation of pus. A careful study of these cases shows them to be those in which suppuration is imminent but not yet absolutely existant. For instance, there may be present a mild degree of swelling, with an albuminous exudate, all of which may be resorbed without pus formation. Whether we are to look with favor, or not, upon Metschnikoff's explanation of the disappearance of the relatively few bacteria present in such cases, is a matter which I hesitate to discuss; though, for my own part, I certainly think it offers a most attractive and reasonable explanation. Virchow's vivid picture of the "battle of the cells" surely loses nothing from Metschnikoff's treatment of the same subject, and phagocytosis is not yet disproved.

Virchow introduced the term "metastatic," and taught us what metastatic abscesses are, and the embolic process by which they are formed. The term loses nothing of its significance in the light of recent enlargements of our knowledge. The emboli which cause them are themselves infected, or even individual germs may be transported *via* the blood current as most minute emboli, and the only uncertain or unappreciated feature of this part of the subject is the determination of *why* minute and metastatic abscesses appear in one place and not in another. This may be, in some cases, the result of pure accident. In general, it compels us to fall back upon the explanation of a *locus minoris resistentiæ.* This may be some mechanical lesion, perhaps one too minute for our vision, or some fracture or previous inflammatory focus. Points of least resistance certainly do exist, though what so constitutes them may be beyond our ken. No one can long study minute pathology without being convinced that there may occur a certain vulnerability of tissue, so to speak, for which we can offer no suitable explanation. The communication of contagion from one person to another is common evidence of this fact. Tissues, then, which suppurate are vulnerable in this respect: they succumb, not having the power to resist infection; that is, the invasion of their bacterial enemies, and pus is the evidence of the conquest of vegetable cells over animal cells.

The matter is a difficult one to treat of. We have forms, and forms of pus-formation. As Grawitz has shown, we have to deal with pus under at least four apparently different circumstances:

1. Cases of typical pyemia.

2. Abscesses at points of least resistance.

3. Apparently spontaneous suppurations, *e. g.*, acute osteomyelitis.

4. Abscesses at points where there has been previously an inflammation.

He and Rinne have pointed out that the localization of pyogenic cocci is an affair of local determination, of interference with absorption, of chemical poisoning (through the circulation), of peculiar disposition of tissues (peculiar to various ages), of local ischemia, etc.; in other words, that by existing local irritations, by beginning inflammatory disturbances, or by regenerative cell proliferations, in spite of previously held opinions, the metastatic grouping of cocci is absolutely prevented.

Rinne divides suppurations into two groups:

1. Those determined by bacteria of peculiar activity, whose attack upon the organism is vigorous, *e. g.*, tuberculosis, actinomycosis, and epidemic cerebro-spinal meningitis, are caused by such organisms as seem to have a peculiar virulence, aside from any pyogenic properties.

2. Those determined by the members of the now well-known group of pyogenic cocci, particularly including staphylococcus and streptococcus.

We are confronted in this study by a most significant fact, which is very difficult of explanation. We have experimental proof that pyogenic cocci may be introduced into the tissues in no inconsiderable number—the same thing occurring every day in many accidental ways—that they may even be found circulating in the blood, without calling forth either suppuration or notable inflammation. According to the researches of Wyssokowitsch, they do not escape by the kidneys. What, then, does become of them? It would appear, as Grawitz says, that (*a*) they are dissolved, and disappear in the blood and other fluids; or that (*b*) there is an active conflict between them and the cells, a struggle for existence, which Virchow, as stated, has already called "the battle of the cells." The best known defender of the first view is Baumgarten, while Metschnikoff's name is most prominently associated with the second. Here, again, there is really much to be said on each side, and there seems to be no reason why each may not be right. According to Grawitz, the cocci usually die in pus after six to ten days, that is at a time when cell activity in the pus has ceased. Beyond a certain point, increase of cocci is impossible in pus, since the fluid becomes a too concentrated albuminoid material for them, just as syrups are too strong sugary solutions for the growth of fermentative and other organisms. On blood-clot they do not grow, though they will on blood-serum. Active penetration of cocci into white corpuscles is out of the question; therefore, when they are found in the interior of leucocytes, the latter must be regarded as the active agents. Certainly, cocci are found inside the pus-cells, for anyone may see them there, and pus-cells, if we know anything about pus, were many of them originally leucocytes. Certainly, too, one cannot say which he has to deal with, when isolated, a pus-cell or a leucocyte, unless he finds it containing one or more cocci imbedded in it.

If, then, in this battle of the cells when once infection has taken place, the parasites are victorious, whether from overwhelming numbers, or from finding their enemies weakened from disease, then the infection of the surrounding tissues extends, and metastatic abscesses may finally or speedily result in the patient's death. On the other hand, if the tissue elements can successfully resist, then the battlefield is surrounded by a wall of young cell elements, which are very rapidly proliferated, and we have only a local abscess, in whose walls certainly takes place some of the phagocytosis which Metschnikoff has so successfully described. The course of that particular suppurative process is henceforth determined, not so much by production of some ptomaine as by the reaction of the cell elements most concerned. So soon as the bacteria die or are killed, in case the pus has not been evacuated, the pus-cells undergo fatty metamorphosis, gradually disappear by absorption, or perhaps caseate in part; for an indefinite time there remains a concealed scar to mark the site of the old battleground, and finally all local and general damage is repaired.

Herein, too, we see the difference between recent and old abscesses, in respect to the so-called "pyogenic" membrane. The protective cell elements thrown out about an infected spot, as alluded to above, are a matter of hours, or, at most, of a few days existence. No time is afforded for organization, nor is it desired. They are meant to serve only as a temporary barrier. Consequently, in an acute abscess we must not expect to find any such membrane; and, if it is folly to look for it, how much more so to describe it, as some have attempted to do. Only in the subacute abscess, or for some weeks pent-up collection of pus, can we find anything approaching it. But it is in the cold abscess, the long-existing one, *par excellence*, that we find the pyogenic membrane, so-called; that is, a membrane or lining which can be peeled or stripped off, though it is a sad misnomer to call it a *pyogenic* membrane since it is anything but this. It is the result of the organization and condensation of this zone of protective cell elements, which were thrown out when the infection and the encroachment were new, which was supposedly intended to be temporary, but has persisted as long as that encroachment from which it was originally intended to protect, and which has grown old and hardened in this service. It is no more pyogenic, in the strict sense of the term, than it is chromogenic, and it should be dropped for a better term. If we must have a descriptive name for this membrane, and it is well that we should, I would like to suggest that we call it *pyophylactic*, as indicating clearly its function if not its appearance.

Pus proper comes to our notice in four ways:

1. In circumscribed subcutaneous collections of new formations —*acute abscesses.*

2. From the surfaces of shut sacs and cavities—*empyemas.*

3. On exposed tissue surfaces and granulating wounds—*pyorrheas.*

4. In the shape of *purulent infiltration* of subcutaneous tissues, more or less deeply occurring.

Pus proper, then, is a mixture of originally good cellular materials gone to the bad, suspended in fluid more or less albuminous, and containing at times adventitious substances, like biliary or hæmic coloring matter, etc.

When pus-cells have undergone fatty changes, when vital activity of all cells, parasitic or otherwise, has subsided, and when more or less of the fluid portion has been absorbed, leaving more concentrated, semi-fluid or solid residue, and when this has perhaps undergone caseous degeneration, then this material is not pus in the sense in which I am using the term, whatever it may have been originally. So long as it has the general appearance of pus, I would suggest for it the name *pyoid* or *puruloid.* When it is caseated, or is so thick that it does not flow, I would suggest that we then call it *archepyon*, that is to say, "originally pus." I introduce these new names to you with considerable hesitation and with becoming modesty, yet I am convinced that if we had names for the different materials, or the different conditions of the same material, it would conduce to clearer notions concerning the substances themselves.

CONCLUSIONS.

1. Inflammation is, in effect, a disturbance of cell nutrition, along with cell proliferation, causing a recurrence to the embryological condition of certain of the cells of the tissue most involved.

2. This embryonal condition means a reversion to the form of those medullary or indifferent corpuscles, from which in the beginning of its normal development the tissue was built up.

3. Congestion, and even stasis, though they precede inflammation, do not necessarily cause it. They may subside before cell nutrition has had time to suffer. They may simply cause temporary cell activity.

4. Medullary, indifferent, or embryonic cells arise not only from the recognized cells of the tissue, *i. e.*, its active protoplasmic elements ; it is probable that the intercellular or basis substance, which was originally produced from embryonic tissue, may again give rise to them.

5. When such new formed embryonic cells advance again to the condition of basis substance, much of the inflammatory new formation has subsided. When with this is coupled restoration to the circulation of exuded fluids and such red and white blood corpuscles as are capable of return, and when all other newly-formed cells are liquefied and absorbed, then the process of *resolution* is complete.

6. When both inflammatory and new embryonic cells establish a reticular intra-connection, then we have a true hyperplasia.

7. When into this collection of cells, parasitic vegetable cells (bacteria) are intruded, no matter how, blood-vessels break assunder, basis substance is dissolved, the individual animal cells are attacked, and these are now suspended in an albuminous fluid and represent pus corpuscles, and we have now a collection of pus.

8. Pus-cells are no longer fit for any useful purpose, but constitute a source of offence. Henceforth they are treated as foreign bodies, of which the tissues endeavor to rid themselves at once. Nature extrudes them in the direction of least resistance, and hence we have the well-known phenomenon of the "pointing" of the abscess.

9. So far as we can learn, bacteria, and bacteria alone, can determine, in the human body, such a series of changes as lead to the formation of pus, *i. e.*, pus within the meaning to which I have endeavored to confine it. Whatever results may follow experimental introductions of a few chemicals into the tissues of some of the lower animals, such experiments find no parallel in our clinical experiences. Moreover, as stated above, the product of such experiments is not pus, but puruloid ; it lacks the essential pathogenic and noxious elements of pus,—the microörganisms which confer upon it its infective and toxic properties.

10. We are then prepared to make the brief and explicit statement that *clinically*, at least, we have no suppuration except such as is produced by bacteria ; in other words, that pus is a product of parasitic origin.

Upon motion of Dr. F. H. POTTER, a vote of thanks was tendered the President for his address.

Beginning the discussion, Dr. BERGTOLD described the process of inflammation set up under the finger-nails by the use of arsenic in taxidermy. He thinks the purulent material resulting therefrom contains no bacteria. He agreed with Dr. Park's definition of pus.

Dr. S. Y. HOWELL believed Dr. Park to be correct.

Replying to a question of Dr. Pryor's, as to bacteria in pleuritic effusions, Dr. PARK gave some of the results of Fraenkel's investigations.

Dr. ROCHESTER raised the question why two fluids, having the same physical properties, should not both be called pus. Dr. Park said a fluid containing bacteria, and, therefore, septic and infectious, should be called pus, while material resembling it, but not septic noi infectious, should be called puruloid on the grounds given in his paper.

A candidate for membership presented a spindle-shaped tumor of the umbilical cord, the size of a child's fist. It was supposed to be a myoma.

Another candidate gave an infant's heart having a patent foramen ovale. The child died asphyxiated four hours after birth, and was the third child dying from such a condition, born of the same mother.

In the business meeting occurred the annual election of officers, which resulted as follows:

*President*—Dr. DeLancey Rochester.
*Vice-President*—Dr. Charles G. Stockton.
*Secretary and Treasurer*—Dr. Eugene A. Smith.

